# Cholera at Buffalo

**Published:** 1852-10

**Authors:** 


### EDITORIAL DEPARTMENT.

*Cholera at Buffalo.* — In our last issue we stated that epidemic cholera was then prevailing to a considerable extent in the city. Cases of the disease began to make their appearance early in July, and continued to increase in number during that month. As no reports were made to the Board of Health, the number of cases occurring in July cannot be ascertained. During the month of August there were reported, by physicians and sextons, 326 deaths from cholera. This does not embrace cases of death by cholera infantum, of which 128 were reported during the month of August. The epidemic continued with unabated severity up to about the middle of Sept. At this time the weather became cold; a rain storm of several days' duration occurred, which was followed by fine autumnal weather, and the disease rapidly subsided. At the present time, September 25th, the city is nearly free from cholera; and, excepting some cases of dysentery, there is very little sickness. The number of deaths, reported by physicians and sextons, from September 1st to 12th, inclusive, was 300. The whole number of deaths reported from August 1 to September 12, thus, amount to 626. Including the unknown number of cases occurring prior to August, the fatality of the disease has been nearly, if not quite as great as in 1849. In that memorable season the number of deaths reported between June 25th and September 7th, was 858.

The duration of the epidemic during the past season, was about the same as in 1849, commencing somewhat later, and continuing longer into September, occupying in its career a little over two months.

The disease prevailed most in sections of the city comparatively insalubrious. At first it was most rife at a part formerly known as the "*flats*," the ground being, as the title implies, low, and requiring, for the most part, to be filled up to protect it against inundations from the lake during high winds. During the latter part of the time there was most suffering toward the eastern limits of the city where the ground is low and marshy. Occasional cases, however, occurred in all parts of the city.

The poorer classes of society furnished a large majority of the victims. At a rough estimate, nineteen-twentieths of the fatal cases were among the foreign population, viz., Irish, Germans, and Norwegians.

The tendency to disorder of the digestive system, among all classes, during the prevalence of the epidemic, was observed, as in 1849. Other affections were extremely infrequent. Medical practice consisted almost exclusively in prescribing for diarrhœa; so much so, that to meet with a case which did not involve special attention to the bowels was almost a luxury, by relieving the monotony of the daily duties of the practitioner. It is common to regard the diarrhœa which coexists with a cholera epidemic as denoting a proclivity to the latter form of disease. Whether this view be correct, or not, the safest policy, undoubtedly, is to act upon the presumption of its being so. With medical treatment it is certain that these *premonitory* symptoms, as they are termed, very rarely eventuate in cholera. As respects this point, our own late experience accords with that which we gave in our report of the epidemic in 1849, (Buffalo Med. Jour., Nov., 1849.) Out of the host of cases of diarrhœa which have come under treatment during the past summer, well marked cholera supervened but in a single instance;* and, on the other hand, in the few cases of cholera that have occurred in our private practice, in all the disease was either developed abruptly, or the premonitory symptoms had been neglected.

In treating the prevailing diarrhœa during the past season, we employed anodyne remedies almost uniformly and exclusively, to wit, opium in tincture, or substance, and morphia, given in pretty full doses; repeated according to circumstances, quietude, and care in dietetics being enjoined. This course we found sufficiently reliable, without exposing patients to the risk of ptyalism.

Not having had the misfortune to meet with many cases of fully developed cholera, our experience in its treatment during the past season has been limited. So far as our observations go, they furnish some striking illustrations of the efficacy of morphia, given promptly in pretty large doses, in arresting, at once, the progress of the disease. We have kept notes of some cases treated after this plan which we may report at a future time.

A conclusion at which all practical observers have arrived, with respect to diminishing the fatality from epidemic cholera, is, that it is a disease to be prevented, or arrested very early in its progress, rather than cured. If it advance to what is generally known as the stage of collapse, recoveries, under any plan of management, will be few. The blood sustains lesions which set the resources of therapeutics at defiance. The important question, then, how shall we stay the ravages of this terrible pestilence, involves another question, viz., how is it to be averted or arrested, in individual cases, during the continuance of the epidemic constitution? Facts show that in the vast majority of cases the development of the disease is preceded by ordinary diarrhœa, and that if the prodromic disorder be treated by simple remedies, and proper precautions observed, a person is nearly secure. The disease prevails chiefly among the classes who do not understand the importance of, or who obstinately neglect to resort to prophylectic measures, and it occurs to a very limited extent only among intelligent members of society who appreciate, and are able to avail themselves of the security afforded by seasonable management. Now, if the whole community, during the prevalence of cholera in a place, could be placed on an equal footing with those whose prudence thus renders them almost exempt from a liability to the disease, it is clear that the number of cases would be greatly diminished. How is this to be effected: in other words how is the disease to be averted, or arrested, among

* In this case the patient was an infant.

the class particularly exposed by their want of intelligence, poverty, and social position? The only effectual plan is to institute a system of sanitary surveillance, to be steadily maintained so long as the epidemic constitution lasts. It is not enough to give public advice by handbills, etc. The object can be fully accomplished only by organizing a voluntary police, subdividing a city into wards, and providing for one or two daily visits by a health officer at every house, his duty being to inquire particularly after the health of every inmate, give directions in case of disorder, and supply remedies. If this plan were to be thoroughly carried out, we verily believe it would render the fatality from this disease small in comparison with what it would otherwise be.

We would not be understood by these remarks to undervalue other sanitary measures for preventing cholera and staying its progress. Facts abundantly prove that the special cause inducing the disease is rendered efficiently active mainly by the co-operation of other causes which we can, to a certain extent, determine, and control. The importance of due attention to the latter is obvious. The plan which we have proposed, however, has never, to our knowledge, been fully adopted, and as it is altogether probable that the disease will continue to recur in different parts of our country, it is to be feared there may be future opportunities to test its merits.

On the subject of malaria as an exciting cause of cholera, Prof. Hamilton has drawn up an elaborate paper, contained in the original department of this No., which it is quite unnecessary for us to commend to the careful perusal of our readers.

